# TRPC Channels in the Physiology and Pathophysiology of the Renal Tubular System: What Do We Know?

**DOI:** 10.3390/ijms24010181

**Published:** 2022-12-22

**Authors:** Colya N. Englisch, Friedrich Paulsen, Thomas Tschernig

**Affiliations:** 1 Institute of Anatomy and Cell Biology, Saarland University, 66421 Homburg/Saar, Germany; 2 Institute of Functional and Clinical Anatomy, Friedrich Alexander University Erlangen-Nürnberg, 91054 Erlangen, Germany

**Keywords:** TRPC3, TRPC6, kidney, renal tubular system, transient receptor potential, renal carcinoma, ischemic injury, autosomal dominant polycystic kidney disease

## Abstract

The study of transient receptor potential (TRP) channels has dramatically increased during the past few years. TRP channels function as sensors and effectors in the cellular adaptation to environmental changes. Here, we review literature investigating the physiological and pathophysiological roles of TRPC channels in the renal tubular system with a focus on TRPC3 and TRPC6. TRPC3 plays a key role in Ca^2+^ homeostasis and is involved in transcellular Ca^2+^ reabsorption in the proximal tubule and the collecting duct. TRPC3 also conveys the osmosensitivity of principal cells of the collecting duct and is implicated in vasopressin-induced membrane translocation of AQP-2. Autosomal dominant polycystic kidney disease (ADPKD) can often be attributed to mutations of the *PKD2* gene. TRPC3 is supposed to have a detrimental role in ADPKD-like conditions. The tubule-specific physiological functions of TRPC6 have not yet been entirely elucidated. Its pathophysiological role in ischemia-reperfusion injuries is a subject of debate. However, TRPC6 seems to be involved in tumorigenesis of renal cell carcinoma. In summary, TRPC channels are relevant in multiples conditions of the renal tubular system. There is a need to further elucidate their pathophysiology to better understand certain renal disorders and ultimately create new therapeutic targets to improve patient care.

## 1. Introduction

The transient receptor potential (TRP) channels are key sensors and effectors in cellular adaptation to environmental stress [1]. The 29 channels are subdivided into seven subfamilies. Canonical TRPC1-7, vanilloid-receptor TRPV1-6, melastatin TRPM1-8, ankyrin TRPA1, polycystin TRPP2/3/5, mucolipin TRPML1-3, and no mechanoreceptor potential C TRPN1 subfamilies are defined [2]. TRPC channels are tetramers and belong to the nonselective cation channels. They are transiently permeable for both monovalent and divalent cations including Na^+^, K^+^, Ca^2+^, or Zn^2+^. Monomeric subunits assemble to form the tetrameric structure. The subunits can belong to the same or to different TRPC entities. Based on amino acid sequences and functional analogies, three subgroups can be distinguished in humans, which preferably heteropolymerize among themselves—TRPC1, TRPC4/5, and TRPC3/6/7 [3]. Six transmembrane segments (S1–S6) are linked to form a TRPC monomer. The cation-permeable pore is generated by the S5 and S6 segments of each subunit [4]. The regulation of the gating activity is subject to a diversity of mechanisms, often related to G_q/11_- and receptor tyrosine kinase-associated phospholipase C pathways, G_i_ and G_o_ proteins as well as intracellular Ca^2+^ stores [3,5]. The protein kinase C can for example directly activate TRPC channels through phosphorylation [6]. Both, mechanical and oxidative stress can also alter TRPC gating behavior [7,8,9]. DAG sensitivity (1,2-diacylglycerine) is a key feature enabling phospholipase C pathways to impact the activity of TRPC3, TRPC6, and TRPC7 [10]. Furthermore, low basal concentrations of DAG can potentially be empowered by pharmacological allosteric modulation of TRPC6 to activate the channel [11]. In contrast, TRPC1, TRPC4, and TRPC5 are insensitive to DAG [6]. TRPC4, however, is indirectly activated by phosphodiesterase-5-inhibitors as demonstrated using human embryonic kidney 293 and prostate smooth muscle cell lines. Cyclic guanosine-monophosphate (cGMP) is degraded by the phosphodiesterase-5. When the latter is inhibited, cGMP can stimulate the protein kinase G (PKG) which in turn phosphorylates and activates TRPC4 ultimately resulting in an increased cytosolic [Ca^2+^] [12]. In addition, TRPC channels play a critical role in inflammation. For instance, TRPC6 is upregulated in microglia by amyloid β-protein in a nuclear factor κ-light-chain-enhancer of activated B-cells (NF-κB)-dependent manner [13]. On the other hand, upregulated TRPC6 channels inhibit the signal transducers and activators of transcription (STAT) signaling and promote proliferative and inflammatory processes in tubular cells in diabetic nephropathy [14]. The role of TRPC6 in diabetic nephropathy is reviewed in [15,16]. In bronchial epithelial cells, TRPC6 is overexpressed after lipopolysaccharide (LPS) exposure and subsequent Toll-like receptor 4 (TLR-4)/phosphatidylinositol 3 kinase (PI3K)/ protein kinase B (Akt) signaling. The following TRPC6-dependent activation of ERK1/2, p38 and NF-κB triggers a cytokine-associated inflammatory response [17].

**Figure 1:**
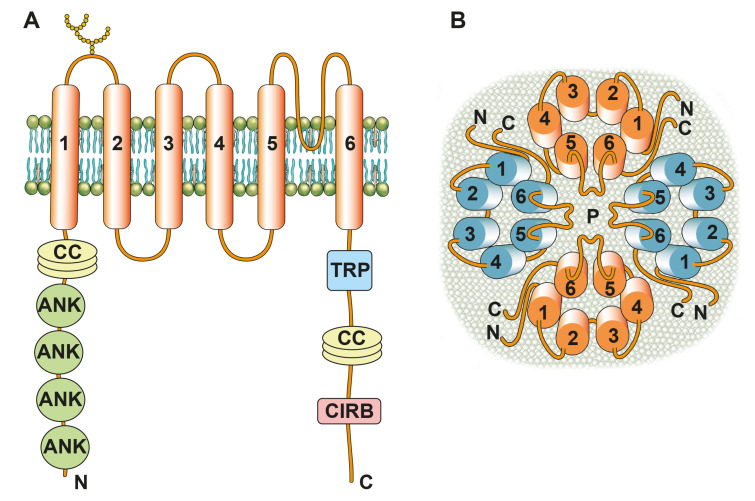
A schematic drawing of the transient receptor potential canonical (TRPC) structure. Six transmembrane segments (numbered 1 to 6) contribute to the formation of a monomer. Both the COOH (C) and NH_2_ (N) terminus feature different domains enabling further channel interactions. These include coiled-coil domains (CC), ankyrin domains (ANK), a TRP box (TRP), a calmodulin, and IP_3_-R binding site (CIRB). Different outer potential glycosylation sites exist and differ among the different TRPC entities [19] ((**A**); inspired from [20]). Four monomers, from the same or different TRPC entities, assemble to form a homo- or heterotetramer. The loops between the transmembrane segments 5 and 6 contribute to the formation of the cation-permeable pore (P) ((**B**); view from above).

Several domains enrich the cytoplasmic TRPC-monomer-termini enabling interaction with a spectrum of molecular players. A coiled-coil domain and four ankyrin domains are localized at the NH_2_-terminus. They are involved in tetramerization of TRPC subunits and thus in the regulation of TRPC channel function. The TRP domain is localized at the COOH-terminus and is the linking site for other TRP-channel isoforms. The COOH-terminus further includes a coiled-coil domain and a calmodulin and IP_3_-R binding site, which regulates store-operated channel activation [7,18] (Figure 1). 

The last domain is involved in the positive regulation of TRPC6 through Ca^2+^/calmodulin (CaM)-dependent kinase II in the cardiovascular system [21]. Elevated cytosolic [Ca^2+^] can activate CaM kinases which in turn may further enhance Ca^2+^ influx through activation of Ca^2+^-permeable channels including TRPC6 [21]. This is a mechanism that can potentially impact both physiological and pathophysiological conditions in different tissues including the renal tubular system. Indeed, several TRPC channels are deeply involved in Ca^2+^ signaling which can result in cell proliferation, cell migration, etc. [18]. Certain TRPC members are also involved in receptor operated Ca^2+^ entry (ROCE) and store operated Ca^2+^ entry (SOCE), with both mechanisms mediating regulated Ca^2+^ influx [18]. Phospholipase C cleaves PIP_2_ (phosphatidylinositol-4,5-bisphosphat) in DAG (1,2-diacylglycerine) and IP_3_ (inositol-1,4,5-triphosphate) which are critical in ROCE and SOCE, respectively. DAG can directly activate members of the TRPC subfamily as previously mentioned, whereas IP_3_ binds to the endoplasmic IP_3_-receptor inducing Ca^2+^ release from the endoplasmic reticulum (ER). The resulting ER depletion is sensed by stromal interaction protein 1 (STIM1) which interacts with Orai1 (calcium release-activated calcium channel protein 1) mediating store-operated Ca^2+^ entry. TRPC1 also plays an important role as store-operated channel while further TRPC channels regulate Orai1 and ultimately SOCE [6,18,22,23]. 

**Figure 2:**
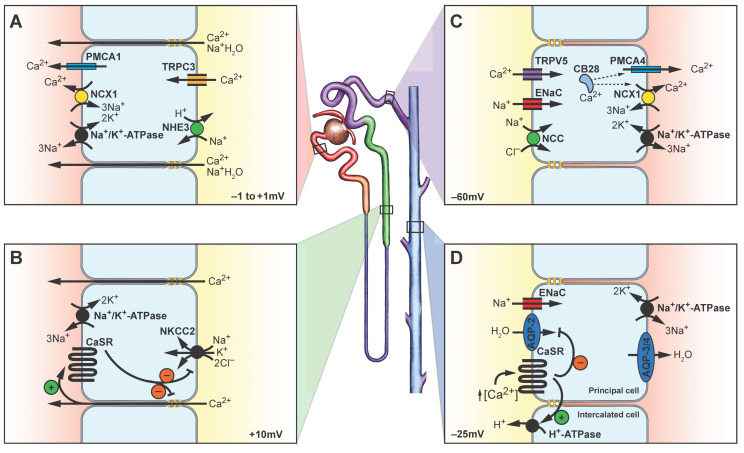
A schematic drawing of the renal tubular system. The glomerulus (brown) produces a primary urine that flows into the proximal convoluted tubule (red). Then, it reaches the proximal straight tubule before ending up in the loop of Henle, which is composed of a thin (dark blue) and a thick (green) part. The latter drains the urine into the distal convoluted tubule (purple) before reaching the collecting duct (light blue) via the connecting tubule, which is not specifically displayed here [24]. The magnifications detailed in (**A**–**D**) reveal physiological mechanisms of the tubular Ca^2+^ reabsorption. Approximatively 98% of the filtered Ca^2+^ is reabsorbed. About 65% are reabsorbed in the proximal tubule, 25% in the thick ascending limb, and 8% in the distal and connecting tubule. The collecting duct plays a negligible role in this context. Most of the Ca^2+^ reabsorption in the proximal tubule is of paracellular nature and partly driven by the transcellular Na^+^ reabsorption mediated by the apical sodium/proton exchanger 3 (NHE3) and the basolateral Na^+^/K^+^-ATPase. Transcellular pathways and the roles of TRPC3, the plasma membrane calcium ATPase 1 (PMCA1), and the Na^+^/Ca^2+^-exchanger 1 (NCX1) are discussed below (**A**). The thick ascending limb features a regulated paracellular Ca^2+^ reabsorption which is driven by the Na^+^ reabsorption which is mediated by the Na^+^/K^+^/2Cl^—^ cotransporter (NKCC2) and the Na^+^/K^+^-ATPase. In terms of a negative feedback-loop, reabsorbed Ca^2+^ can activate basolateral calcium sensing receptors (CaSR) which in turn decrease the Na^+^ reabsorption and the paracellular claudin-mediated Ca^2+^ reabsorption (**B**). In the distal and connecting tubules, however, Ca^2+^ reabsorption is of a transcellular nature. Sodium enters the cell through the epithelial Na^+^ channel (ENaC) or the Na^+^/Cl^—^ cotransporter (NCC) and leaves it on the basolateral side using the Na^+^/K^+^-ATPase. The latter drives the Na^+^/Ca^2+^-exchanger 1 (NCX1) which shares the function of basal Ca^2+^ discharging with the plasma membrane calcium ATPase 4 (PMCA4). Apical Ca^2+^ entrance occurs using the vanilloid transient receptor potential 5 channel (TRPV5). Transcellular transport is mediated by calbindin D-28k (CB28) (**C**). Luminal Ca^2+^ concentration is sensed by calcium sensing receptors (CaSR) in the collecting duct. Their activation leads to the inhibition of aquaporin-2 (AQP-2)-mediated H_2_O reabsorption in the principal cells and activation of the H^+^-ATPase in the intercalated cells with subsequent urine acidification ultimately reducing the probability of crystal precipitation. Basolateral H_2_O transport occurs through aquaporins-3 and -4 (AQP-3/4) (**D**). Inspired by [25].

The nephron is the functional unit of renal physiology. The nephron is subdivided into a renal corpuscle—the compound of glomerulus and Bowman’s capsule—and a tubular system composed of proximal, intermediate, and distal tubules that drain into a collecting duct. The primary urine is gained at the glomerular filtration barrier and transformed into a secondary urine through many different reabsorbing tubular mechanisms [24] (Figure 2). 

The renal tubular system underlies several hormonic loops such as the renin-angiotensin-aldosterone-system (RAAS) or the vasopressin system and is essential in acid–base and ion–water homeostasis [26,27]. From a pathophysiological point of view, substantial damage can arise from impaired tubular function. For instance, highly metabolic-active proximal tubule cells can be damaged by multiple conditions leading to acute kidney injury [28,29]. Additionally, a disturbed Ca^2+^ reabsorption can promote crystal precipitation with concomitant nephrolithiasis leading to inflammation and fibrosis and eventually resulting in chronic kidney disease [30,31,32]. Furthermore, malignant processes such as renal cell carcinoma originate from the tubular system [33]. In short, the tubular system is not only crucial for renal physiology, but also for a multitude of pathophysiological processes that remain challenging today, as most of them cannot yet be treated satisfactorily.

Ever since evidence suggested that mutations in the *TRPC6* gene could lead to focal and segmental glomerulosclerosis (FSGS) [34], the research in renal TRPC channels has dramatically increased. The relationship between TRPC6 and glomerular permselectivity and its involvement in renal proteinuric disorders has been of special interest [35]. However, TRPC6 is not only found in the glomeruli, but also in the renal tubular system [36]. In the last few years, more and more studies have investigated TRPC channels—especially TRPC3 and TRPC6—in the renal tubules. However, the role of TRPC6 in tubular physiology remains insufficiently studied. It has been suggested that TRPC6 could promote flow-stimulated generation of endothelin-1—an autocrine inhibitor of sodium and water reabsorption—in cortical collecting ducts [37,38]. The role of TRPC channels in glomeruli and their involvement in proteinuric, diabetic, and chronic kidney diseases but also in renal fibrosis is summarized elsewhere [15,16,35,38,39]. We present here a summary of the implication of TRPC channels in both the specific physiology and the specific pathophysiology of the renal tubular system. 

## 2. TRPC6 Is a Controversial Player in Tubular Cells Experiencing Ischemia-Reperfusion Injuries

Several conditions, such as acute hemorrhage or toxic shock, can cause renal ischemia-reperfusion injury (RIRI). RIRI is characterized by massive tissue damage and is a frequent cause of acute kidney injuries [40]. Acute kidney injuries can be defined as a 1.5-fold baseline increase in serum creatinine over the preceding seven days [41]. The generation of reactive oxygen species (ROS), Ca^2+^ overload, and immune responses are key factors in the promotion of tubular damage in acute kidney injury following RIRI [42]. Tubular injury is also considered as a driving force towards chronic kidney disease (CKD) [43]. Proximal tubular cells have an active metabolism and are therefore especially endangered by the oxidative stress that can occur during ischemia-reperfusion (I/R) [29,44,45,46,47,48]. Interrupted perfusion, for instance caused by thromboembolism, leads to a transition from aerobic to anaerobic cell metabolism and concomitant impaired ATP production (adenosine-triphosphate). This in turn is associated with acidification and an intracellular [Na^+^] and extracellular [K^+^] increase. Subsequently, membrane depolarization with compensatory Ca^2+^ influx induces the activation of proteases which contribute to the resulting cell death. Sudden reperfusion with concomitant reoxygenation creates a massive release of reactive oxygen species (ROS) [49]. ROS comprise free radicals, oxygen anions, and hydrogen peroxide [50]. The oxidative burst heavily damages tissues—cytoprotective ROS scavengers being disabled after ischemia-reperfusion—leading to different forms of cell death including apoptosis, necrosis, necroptosis, pyroptosis, ferroptosis, etc. [51]. Both ischemia and reperfusion contribute to the heavy tissue damage [51,52,53,54,55,56,57,58,59,60,61,62,63]. In 2013, a bioinformatic analysis of rat samples showed that TRPC6 was upregulated in RIRI-damaged tissues compared to the control [64]. Further investigations supported the upregulation of TRPC6 in RIRI [65]. The literature is not concordant, as the downregulation of TRPC6 has also been observed in I/R tissue compared to sham tissue [66,67]. ROS—released during RIRI—are involved in both the regulation of TRPC6-expression and -gating activity as well as the initiation of autophagy [8,50,68]. The altered Ca^2+^ signaling, which is mediated by the redox-sensitive TRPC6 channel, is involved in ROS-caused renal injury [8,9]. Autophagy is a dynamic recycling cellular process decomposing cell components in a lysosomal environment which is induced by the formation of autophagosomes in response to oxidative stress in ischemia-reperfusion injuries [69,70,71,72,73]. It is rapidly enhanced after reperfusion, ultimately deferring the increase of apoptotic activity in RIRI. The densitometric analysis of microtubule-associated proteins 1A/1B-light chain 3 (LC3-II/LC3-I), p62 and B-cell lymphoma 2 (Bcl-2)/Bax blots combined with hematoxylin and eosin, and terminal deoxynucleotidyl transferase dUTP nick end labeling (TUNEL) staining revealed that the inhibition of autophagy using the PI3K-inhibitor 3-methyladenine (3-MA) aggravated tissue damage [40]. These results contribute to the concept of a cytoprotective function of autophagy in RIRI.

Hou et al. suggested that TRPC6-mediated Ca^2+^ influx regulates autophagic flux in proximal tubule cells undergoing oxidative stress through H_2_O_2_ exposure. Indeed, both basal as well as oxidative stress-associated autophagy can be decreased or increased by respective overexpression or silencing of TRPC6 [69]. Furthermore, apoptosis is attenuated in TRPC6 SAR7334-silenced proximal tubule cells after H_2_O_2_ exposure. Additionally, the mitochondrial permeability transition positive cells—hallmarks of ROS injury—were significantly diminished after SAR7334 silencing of TRPC6. Additional evidence was obtained suggesting that post-oxidative stress apoptosis is attenuated by increased autophagic flux through TRPC6 silencing [69]. Moreover, the involvement of the PI3K/Akt/mTOR and ERK1/2 pathways in TRPC6-driven autophagy inhibition after ROS exposure has been demonstrated. A mechanism of TRPC6-mediated Ca^2+^ signaling resulting in phosphorylation of Akt and ERK preventing autophagy but enhancing apoptosis in response to ROS, was proposed. However, contradicting evidence has appeared indicating that cell autophagy is promoted by I/R injury and accelerated in tubular epithelial HK-2 cells overexpressing TRPC6 [74]. The suggestion of Hou et al. was not refuted by Shen et al. who observed that apoptosis is not affected by TRPC6 [75]. In turn, they suggested that TRPC6 may inhibit necroptosis and thereby play a protective role in RIRI [75]. Restoration of TRPC6 expression—which was reduced after ischemic injury—was awarded a role in alleviating RIRI-induced acute tubular injury after erythropoietin premedication in collecting ducts [38,76]. Moreover, necrostatin-1, a receptor-interacting protein kinase-1-inhibitor (RIP1), was shown to alleviate RIRI; an effect which may be mediated by the hypoxia-inducible factor-1α (HIF-1α/miR-26a/TRPC6/poly (ADP-ribose) polymerases 1 (PARP1) pathway [67]. Shin et al. showed that L-ornithine-dependent activation of the calcium sensing receptor can decrease ROS generation and prevent H_2_O_2_-induced necrosis through TRPC-dependent ROCE in proximal tubular cells and thereby alleviate acute kidney injury [77]. Interestingly, another recent study showed no difference in renal function and tubular damage among TRPC6^-/-^ mice, TRPC6-inhibitor treated mice, and wild type mice after RIRI [42]. TRPC6 inhibition did not impact the short-term outcome of acute kidney injury [42].

Aside from investigating the role of TRPC6, the function of Zn^2+^ in RIRI was also studied. Zinc is a trace element which is essentially involved in multiple physiological processes of the organism [78]. TRPC6 markedly contributes to transmembrane Zn^2+^-transportation and Zn^2+^ itself plays a key role in autophagy [66,79,80,81]. The Zn^2+^ content is increased in cells with ischemia and reperfusion. TRPC6 knockdown or TRPC6 overexpression in oxygen-glucose deprived and reoxygenated (OGD-R) human kidney-2 (HK-2) cells would respectively lead to a decrease or an increase in Zn^2+^-flow—the latter concomitantly with an augmented autophagic flux. Zn^2+^ significantly ameliorated the viability of OGD-R HK-2 cells and decreased the controlled necrosis rate [74]. A recent study revealed the role of TRPC6 and Zn^2+^ in inhibiting pyroptosis of tubular epithelial cells and thereby attenuating RIRI [66]. Pyroptosis is a form of programmed necrotic cell death which is induced by NLRP3 (nucleotide-binding and oligomerization domain-like receptors (NLR) family pyrin domain containing 3) associated caspase-1 activating gasdermin D to form pores in the plasma membrane resulting in proinflammatory cell lysis [82]. Interestingly, insufficient Zn^2+^ levels are thought to mediate activation of NLRP3 inflammasome after ROS exposure and subsequently induce pyroptosis [66,83]. Both animal RIRI models and OGD-R HK-2 cells were used to provide evidence that TRPC6 inhibition augmented pyropoptic activity and exacerbated renal injury in RIRI. Zn^2+^ influx and upregulation of the zinc finger protein A20 inhibiting the activation of NF-κB, which plays a key role in NLRP3 activation, seem to control the cytoprotective and antipyropoptic effects of TRPC6 in RIRI [66,84]. 

In summary, we can conclude that TRPC6 is involved in Ca^2+^ and Zn^2+^ signaling in RIRI. The relevant literature is not concordant on the function of tubular TRPC6 in RIRI. However, Ca^2+^ entry channels such as TRPC6 may have dual roles in renal epithelial cells [85]. Further studies are needed to clarify this discussion in the context of RIRI. A better understanding of the function of TRPC6 in RIRI is important as it may lead to targeted drug development.

## 3. TRPC6 Drives Tumorigenesis and the Progression of Renal Cell Carcinoma 

Unsettled Ca^2+^ signaling is often involved in tumorigenesis [86,87]. Renal cell carcinoma (RCC) is a very common cancer affecting the kidney [88]. More than 300,000 new cases of RCC are reported worldwide yearly [89,90]. Different subtypes of renal cell carcinoma exist. The clear cell entity (70–80%) is the most common type of RCC, directly followed by the papillary type (15%) [91,92]. Both are originated from the proximal tubule [33]. From a biological and clinical point of view, clear cell renal cell carcinoma (ccRCC) and non-ccRCC are completely different pathologies and, therefore, so is the respective tumor-specific therapy. The single curative therapy in many localized cases is often surgical removal. However, studies indicate that 30% of patients who had been considered healed suffered a relapse [88]. Classical chemotherapy or radiotherapy treatments are not able to be used to treat RCC [90]. The focus needs to be placed on specific tumor biology, microenvironments, or vascularization [90]. Unfortunately, the exact pathogenesis of the different renal cell carcinoma types is not well understood. Nevertheless, the Ca^2+^ permeable TRPC6 channel is supposed to be implicated in receptor-operated Ca^2+^ entry of RCC cells. In addition, TRPC6 expression is by far the most increased compared to TRPC3, TRPC4 or TRPC5 expression in ccRCC tissue [93]. Immunohistochemistry has shown that TRPC6 reactivity was significantly stronger in RCC tissue compared to healthy tissue [94]. The tumor nuclear grading according to Fuhrmann was positively correlated with the amount of detected TRPC6, suggesting a significant function of TRPC6 in tumorigenesis and tumor progression [94]. Song et al. investigated the effects of inhibiting TRPC6 in ACNH cells—a cell line initiated in 1979 from the malignant pleural effusion of a 22-year-old man in the context of a metastatic renal cell carcinoma [95]—and revealed a significantly decreased hepatocyte growth factor-induced (HGF) cell proliferation. Additionally, TRPC6-mRNA inhibition via siRNA3 transfection led to an increased transition time through the G_2_/M phase of mitosis in ACHN cells, eventually providing sufficient time to ensure efficient DNA-repair machinery [94]. In general, TRPC6-gated Ca^2+^ influx has been shown to be critical in the transition of the G_2_/M phase in several different cell types [96]. HGF is a pleiotropic glycoprotein—able to increase TRPC6 expression in ACHN cells—that stimulates the c-met signaling which is a major player in tumorigenesis, progression, and vascularization of papillary RCC [92,97,98]. The MET signaling also mediates VEGF resistance in ccRCC [92]. Indeed, in the context of hereditary papillary RCC, a gain-of-function mutation of the HGF-tyrosine kinase membrane receptor MET (mesenchymal-epithelial transition factor) leads to uncontrolled cancer-promoting effects [99]. Subsequently, Kim et al. investigated the relevance of the lysine-deficient protein kinase 1-promoted (WNK1) TRPC6-NFAT (nuclear factor of activated T-cells) pathway in the development of ccRCC. WNK1 controls the tubular electrolyte homeostasis. Therefore, it regulates the distribution of several ion channels and transporters through various signaling cascades involving effectors such as the STE20-proline alanine rich kinase (SPAK) and oxidative stress responsive kinase 1 (OSR1) but also the mitogen-activated protein kinase (MAPK) [100]. Hence, the impairment of WNK1 function can lead to pseudo hypoaldosteronism type two [101,102]. Furthermore, evidence has emerged uncovering the key role of WNK1 in tumorigenesis [103]. In the context of ccRCC, upregulated WNK1 stimulates the phosphatidylinositol 4-kinase IIIα (PI4KIIIα) enzyme, which controls the phosphatidylinositol-4,5-diphosphate-dependent PLC-β signaling leading to DAG-mediated activation of TRPC6. The WNK1-TRPC6 pathway activates the NFATc1 signaling which in turn has been suggested to enhance WNK1 and TRPC6 expression in ccRCC cell lines. Consistent with these reports, the c-Myc gene, which is regulated by NFATc1 signaling is often overexpressed in RCC [104]. Furthermore, Kim et al. showed that the WNK1-activated TRPC6 is an important player in receptor-operated Ca^2+^ influx in ccRCC cell lines such as Caki1 and ACHN. Functional analysis has revealed that the colony-forming ability of ACHN and Caki1 cells was reduced in knockdown models of TRPC6, WNK1, and PI4KIIIα. The number of cells was also decreased. Inhibition of the TRPC6-NFATc1 pathway markedly diminished the survival as well as the proliferation of ccRCC cells. These results, among others, support the suggestion that the WNK1-driven TRPC6-NFATc1 pathway is a key component in the proliferation and migration of ccRCC cells [93]. In contrast, studies on human metastatic renal cell carcinoma cultures have demonstrated that TRPC6 is involved in SOCE and that SOCE inhibition did not impact cell proliferation [105]. In addition to TRPC6, TRPC1 deserves to be mentioned in the context of tubular players in RCC. TRPC1 is crucial for the polarity and the directionality of migrating cells including cancer cells [18]. A recent study investigated the TRPC1 expression in ccRCC tissue and demonstrated a positive correlation between the TRPC1-expression level and the tumor grade. The authors postulated that TRPC1 might enhance cell proliferation via Ca^2+^ entry and Ca^2+^-NFATc3 signaling pathways leading to ccRCC growth which is concomitant with higher tumor grading. However, the relevance of TRPC1 was limited to biomarking TNM stages and indicating long-term prognosis of RCC [106]. 

In summary, TRPC6 is a critical factor in tumorigenesis and tumor progression of different RCC entities [93]. Inhibitors of the downstream effector MAPK of WNK1 have been proposed for the treatment of RCC [107]. Similarly, WNK1 or TRPC6, but also MET in the papillary entity [92], may be new targets in the antiproliferative therapy of RCC.

## 4. TRPC3 Is a Cytoprotective Key Player in Ca^2+^ Reabsorption of the Proximal Tubule 

**Figure 3:**
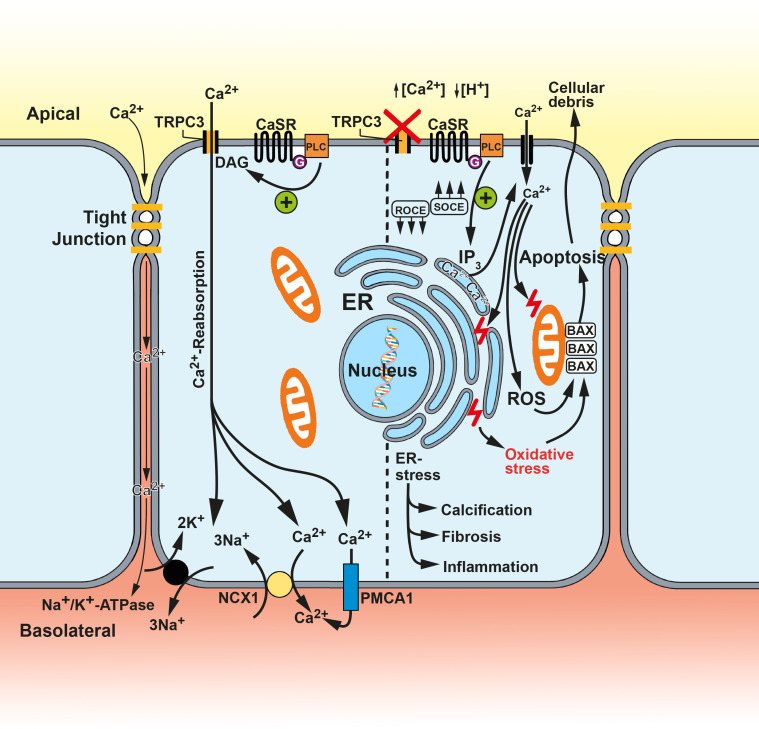
The suggested role of TRPC3 in the proximal tubule cell. The cell is divided in two halves. The left half shows the proposed physiological function of TRPC3 in the proximal tubule. Luminal Ca^2+^ activates the calcium sensing receptor (CaSR), which triggers in turn the G-protein associated phospholipase C pathway. DAG is generated and increases TRPC3 activity. Receptor-operated Ca^2+^ entry (ROCE) results. Basolateral players including the plasma membrane Ca^2+^-ATPase 1 (PMCA1) and the Na^+^/Ca^2+^-exchanger 1 (NCX1) mediate the basal Ca^2+^ export. The right half shows a TRPC3-deficient proximal tubule cell which is exposed to alkaline hypercalciuric conditions. SOCE pathways outweigh ROCE pathways. Then, ER stress and ROS generation follow, along with subsequent calcification, inflammation, fibrosis, and apoptosis. Cellular debris accrue and promote stone formation which in turn aggravates the tubular damage. TRPC3 is suggested to contribute to prevention of such exacerbated luminal Ca^2+^ concentration and subsequently to attenuation of eventual cellular damage. Inspired by [31,109].

TRPC3 is expressed in the proximal tubule and the collecting duct [31,108,109,110]. Approximatively 65–70% of the tubular calcium reabsorption is performed in the proximal tubule [111]. Paracellular mechanisms predominate. A difficulty to explain mechanisms resolving a sudden increase in luminal [Ca^2+^], has remained [109]. For this reason, an inducible transcellular pathway might be superior to simple paracellular osmotic and diffusion processes. The apical compound of calcium sensing receptors (CaSR)—a class three G-protein-coupled receptor—and TRPC3 has been proposed to play a critical role in transcellular Ca^2+^ reabsorption in proximal tubule cells [109,112]. The CaSR—expressed in the gut, kidneys, and parathyroid gland—is a main component of extracellular Ca^2+^ homeostasis and can activate both SOCE and ROCE pathways in the proximal tubule [109,113,114]. Nevertheless, ROCE remains in large parts responsible for Ca^2+^ entry in proximal tubule cells [109]. An alkaline hypercalciuric environment conditions a switch from ROCE to SOCE in TRPC3-deficient proximal tubule cells [31,32,115] (Figure 3). 

The subsequent excess of intracellular [Ca^2+^] can lead to ER stress (endoplasmic reticulum) and ROS production [32,115,116]. NPS-2143, a CaSR inhibitor, reduced SOCE, ROS generation, and ER stress in TRPC3-deficient proximal tubule cells. This argues for a cytoprotective function of the CaSR-dependent TRPC3 activation as the SOCE-associated downstream cascade of injuring events following hypercalciuria is diminished. Excessive luminal [Ca^2+^] can activate the CaSR and initiate the phospholipase C signaling. The resulting DAG messaging can enhance TRPC3-gated Ca^2+^ influx in the proximal tubule. Hypercalciuria and subsequent calcium-phosphate crystal formation in the loop of Henle can thereby be limited [109,117]. Basolateral Ca^2+^-efflux mediators such as the plasma membrane Ca^2+^-ATPase 1 (PMCA1) or the Na^+^/Ca^2+^-exchanger 1 (NCX1) complete the concept of a transepithelial calbindin-mediated Ca^2+^ reabsorption process in the proximal tubule [109] (Figure 3). After oral calcium gluconate administration, TRPC3-gating augmented altering luminal [Ca^2+^] [32]. The critical role of TRPC3 in Ca^2+^ reabsorption is supported by the development of hypercalciuria after TRPC3 knockout [109]. Even though unlikely, CaSR-TRPC3 activation may also reduce luminal [Ca^2+^] by increasing the tight-junction associated paracellular Ca^2+^ permeability [109]. Hypercalciuria is the basement for calcium-phosphate crystal nucleation that displays the preliminary stage of both calcium phosphate and mixed stone formation [117,118,119,120,121]. This sequence—summarized as lithogenesis—is boosted in alkaline milieu. The CaSR can be sensitized by luminal alkalization eventually enhancing an increased Ca^2+^ reabsorption [31,122]. These circumstances were used to facilitate crystal nucleation in experimental designs after acetazolamide administration [31]. Acetazolamide inhibits proximal tubular carbonic anhydrases—essential components in the renal acid–base balance—provoking metabolic acidosis with concomitant tubular alkalization and facilitated crystal formation [123]. Subsequent tubular crystal uptake can activate the NF-κB—NLRP3—IL-1β pathway triggering IL-6 and TGF-β1 secretion which are key factors in advancing renal fibrosis and inflammation [32,124,125,126,127]. Tubular fibrosis and inflammation are exacerbated by TRPC3 knockdown as suggested by histology and increased fibrotic (TGF-β1, FN-1 and SMa) and inflammatory (IL-1β, IL-6, monocyte chemoattractant protein-1 (MCP1), NF-κB and NLRP3) markers [31,128]. The NF-κB pathway is also associated with ER-stress-induced apoptosis [129,130]. Hypercalciuric conditions obtained after the calcium gluconate treatment increased the expression of ER-stress-related genes such as C/EBP homologous protein and M18S [32]. Similarly, the apoptotic activity of proximal tubule cells was increased, especially when TRPC3 was silenced [32]. Disordered extracellular Ca^2+^ concentration can also evoke responsive ROS production driving oxidative cellular injury leading to apoptosis, fibrosis, inflammation, etc., which are concomitant with a decreasing renal function [31,32,131,132,133,134,135]. The resulting cellular debris promote lithogenesis creating a vicious circle [136,137]. TRPC3 may therefore contribute to a postponed and decelerated development of CKD in the context of nephrocalcinosis and -lithiasis [31].

In summary, TRPC3 is critically involved in Ca^2+^ reabsorption in the proximal tubule and its impaired expression can contribute to hypercalciuria and through crystal formation and calcification support both fibrosis and inflammation which can result in acute and chronic kidney disease [115]. Since proximal tubular injury and the crystal formation were exacerbated by TRPC3 deficiency, it is legitimate to attribute a preventive role to TRPC3 in hypercalciuria-induced crystal formation and tubular injury by reabsorption of excess luminal calcium.

## 5. TRPC3 Is Involved in Vasopressin-Dependent AQP-2 Trafficking, Osmosensation, and Ca^2+^ Reabsorption in the Collecting Duct

**Figure 4:**
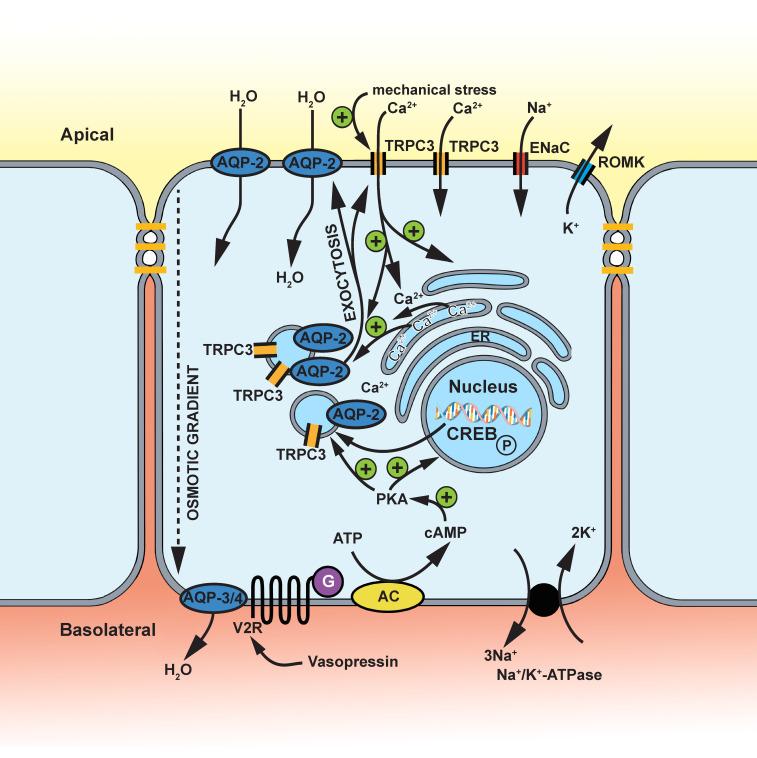
The postulated function of TRPC3 in the collecting duct. Arginine vasopressin stimulates the V2 vasopressin receptor (V2R). A signaling pathway is triggered leading to the generation of cyclic adenosine monophosphate (cAMP) and the activation of the protein kinase A (PKA). Membrane trafficking of aquaporin-2 (AQP-2) and TRPC3, as well as antidiuretic gene expression, is enhanced (CREB or cAMP response element-binding protein). TRPC3 is involved in Ca^2+^ reabsorption and sensing of hypotonicity by initiating Ca^2+^ signaling with direct and indirect stimulation of AQP-2 membrane trafficking. AQP-2 and AQP-3/4 mediate, respectively, apical and basolateral H_2_O flow along the osmotic gradient. The epithelial Na^+^ channel (ENaC) and renal outer medullary K^+^ channels (ROMK) mediate apical Na^+^ influx and K^+^ efflux.

Since Khayyat et al. extensively reviewed the function of TRPC3 in the kidney in 2020 [110], there has been very little research performed on the topic. We will therefore only briefly report the most important findings for the sake of completeness but refer to Khayyat et al. for a detailed review [110]. The collecting duct (CD) is composed of principal cells (PC) and four types of intercalated cells (IC) [138]. While principal cells are important players in the ion–water balance, including Ca^2+^ reabsorption, intercalated cells play a critical role in acid–base homeostasis [138,139]. TRPC3 and TRPC6 are expressed in the principal cells of the collecting duct as aquaporin 2-colocalization (AQP-2) indicates [108,140]. Arginine vasopressin or antidiuretic hormone (ADH) is a major regulator of the ion–water balance in principal cells [141]. Binding to the basolateral V2 vasopressin receptor (V2R)—a G-protein-coupled receptor—results in the activation of the cyclic adenosine monophosphate and protein kinase A (cAMP/PKA) pathway enhancing the membrane trafficking of both AQP-2 and TRPC3 [140,142]. The long duration of the effects of arginine vasopressin is partly attributed to the “non-canonical” β-arrestin1/2-dependent V2R-internalization preserving cAMP-PKA signaling. The latter is instead supposed to be terminated by the endosomal retromer complex—a key component of the endosomal protein sorting machinery [143,144]. Sufficient evidence is presented, ascribing the anticalciuretic effects of arginine vasopressin on TRPC3-positive principal cells which translocate TRPC3 and AQP-2 to the apical membrane after V2R activation enabling an apicobasal Ca^2+^ flux ultimately counteracting calcium crystal formation in times of concentrating antidiuresis [140,145,146]. On the other hand, TRPC3 itself contributes to the translocation of AQP-2 to the apical membrane—as the AQP-2 membrane trafficking likely requires TRPC3-dependent [Ca^2+^]_i_ raise—according TRPC3 an additional crucial role in water homeostasis [146]. Interestingly, apical CaSR activation occurs during antidiuresis—a state characterized by severe urine concentration—and has been proposed to trigger Ca^2+^ reabsorption to limit crystal precipitation. In addition, CaSR signaling reduces the vasopressin-induced AQP-2 membrane translocation within a negative feedback loop allowing formation of a not too severely concentrated urine and preventing nephrolithiasis [147]. Moreover, the collecting duct is not only sensitive to endocrine factors such as arginine vasopressin or aldosterone, but also to alterations of the luminal milieu including changes in osmotic gradients or in flow rate. An increase in [Ca^2+^]_i_ often mediates the adaption of the cellular behavior in matters of water–ion balance, proliferation rates, etc. [148,149,150]. Evidence has been provided suggesting that hypotonicity induces TRPC3-gated Ca^2+^-entry and initiates the downstream osmosensitive signaling cascade which is reinforced by an additional Ca^2+^ release from intracellular stores resulting in cellular behavior adaptation [151] (Figure 4). Nevertheless, it is not exactly clear whether the channel itself is sensor of hypotonicity via its long S3 segment, for example [10], or whether it is only a second player in osmosensitive signaling [110]. In contrast, TRPV4 is a key player mediating the cellular response to alterations in tubular flow which is not affected by osmotic alterations such as TRPC3 [110,152,153]. On the basis of this example, we can retrace the diversity of TRP channels and their need in multiple different roles of sensors and effectors in the context of cellular adaptation to environmental changes.

In summary, TRPC3 is a critical player in the downstream signaling pathway that is triggered by arginine vasopressin stimulation in the collecting duct. Stimulation of TRPC3 could be employed to increase trafficking of AQP-2 mutants causing certain forms of nephrogenic diabetes insipidus. In contrast, TRPC3 inhibition might be critical in reversing excessive water retention which could have clinical benefits in certain conditions including congestive heart failure [151].

## 6. Mitochondrial TRPC3 Drives Detrimental Calcium Uptake and Mediates Cell Proliferation in Autosomal Dominant Polycystic Kidney Disease-like Conditions

ADPKD or autosomal dominant polycystic kidney disease is a genetic disorder that is characterized by multiple bilateral renal cysts resulting in progressive renal failure. It is a very common cause of end-stage kidney disease [154]. In most cases, ADPKD is induced by loss of function mutations affecting the nonselective calcium channels polycystin 1 or polycystin 2 (TRPP2, PKD2, PC2), which are physiologically involved in the regulation of various cellular functions including fluid transport, differentiation, proliferation, cell adhesion, and apoptosis [155,156]. Cellular Ca^2+^ is altered by a decreased channel function resulting in the activation of the adenylate cyclase with generation of cAMP. The latter stimulates the protein kinase A and Ras/Raf/extracellular-regulated signaling kinase (ERK) pathway that promotes cellular proliferation and cystogenesis [157,158,159]. Current understanding of ADPKD pathogenesis is summarized in [155]. Interestingly, oxidative stress and disordered mitochondrial metabolism were linked to the pathogenesis of ADPKD [159,160,161,162]. Both cellular and mitochondrial ROS and calcium mutually interact. Dysregulation of the one might heavily affect the other [163,164]. TRPC3, a critical player in Ca^2+^ signaling, is also found in the inner mitochondrial membrane and can directly interact with NADPH oxidase 2 thereby regulating generation of oxidative agents as shown in cardiomyocytes [159,165,166,167]. The involvement of TRPC3 and TRPC7 as components of TRPP2-mutant channel heteropolymers in receptor-operated Ca^2+^ influx leading to uncontrolled cell proliferation and cystogenesis in ADPKD, has been previously suggested [168]. Transfection of human conditionally immortalized proximal tubular epithelial cells (ciPTEC) and mouse collecting duct cells (IMCD3) with TRPP2-siRNA demonstrated TRPC3-upregulation in ADKPD-like conditions [159]. TRPC3 induced cell proliferation, ERK activation, and mitochondrial dysfunction in interplay with NCX1 upon TRPP2 knockdown. Mitochondrial TRPC3 was also upregulated after TRPP2 knockdown and involved in a mitochondrial Ca^2+^ influx promoting mitochondrial dysfunctions with impaired ROS generation driving cell proliferation [159]. Interestingly, polycystin-2 was shown to regulate calcium homeostasis players including IP_3_-receptors, STIM1, TRPV4, and TRPC1 [156]. However, expression levels of TRPC1, TRPC6, and TRPC7 remained the same upon TRPP2 knockdown [159].

In summary, TRPC3 is upregulated in TRPP2-knockdown cells and impairs mitochondrial calcium which is concomitant with mitochondrial dysfunctions thus driving cell proliferation. TRPC3 has already been proposed as medication strategy in various different diseases [169]. Similarly, TRPC3 may become a new focus in the treatment of the most common hereditary kidney disease—ADPKD. 

## 7. Concluding Remarks

This literature review displays the current knowledge and understanding of the physiological and pathophysiological roles of transient receptor potential canonical channels in the renal tubules of the kidney. In this context, recent research mainly focusses on TRPC3 and TRPC6. Tubule-specific physiological functions of TRPC6 remain unclear [38], whereas TRPC3 is awarded a key role in Ca^2+^ homeostasis in the proximal tubule and collecting duct as illustrated by its Ca^2+^ reabsorbing function. From a pathophysiological point of view, evidence is provided showing the involvement of TRPC6 in renal cell carcinoma emergence and progression. However, its role in ischemia-reperfusion injuries with acute kidney injury is controversial. In contrast, TRPC3 is a protective player in hypercalciuria, while its upregulation and deleterious role have been demonstrated in autosomal dominant polycystic kidney disease-like conditions. All in all, TRPC channels fulfil diverse functions in the renal tubules of the kidney. Their involvement in severe diseases such as acute or chronic kidney damage, but also in renal cell carcinoma is well known. However, some reports need to be interpreted with caution as there have been only few works completed regarding certain topics. More research is needed to further elucidate and substantiate this underappreciated chapter and eventually achieve a better molecular understanding of severe pathophysiological conditions. Subsequent development of further targeted therapies could lead to a better clinical care. 

## Data Availability

Not applicable.
